# Therapeutics

**Published:** 1876-11

**Authors:** 


### I. Therapeutics.

1. *Oleum Terebinthinæ in the treatment of Diphtheria and Tonsillitis.* Peabody. (*Medical and Surgical Reporter*, September 9.)

P. advocates the above remedies both in tonsillitis and diphtheria. In one hundred and twenty-four cases of tonsillitis and ten of diphtheria treated in this way during ten years, the results were uniformly successful. He considers the turpentine stimulant an antizymotic in diphtheria. The following prescription is advised:

℞ Ol. Terebinthinæ ........ ʒ ij

Pulv. Potass. Chloratis ........ ʒ ij

" Sacch. Albi ........ ℥ ss

" Acaciæ ........ ℥ ss

Aquæ ........ ℥ v

M. Sig. Shake well. Tablespoonful each hour or two until inflamma tory symptoms subside, then less often.

In severe cases, if the patient is not relieved inside of twenty-four hours, forty-eight grains of sulphate of quinine are to be added to the emulsion of turpentine. To be taken as before—each two hours, or alternately with twenty drop doses of tr. muriate of iron each two hours. J. S. K.

2. *Local Use of Cold in Abdominal Inflammations.* Eads. (*Norwich Medico-Chirurgical Society.*)

E. strongly advocates the local use of cold to the abdomen in abdominal inflammations, if agreeable to the patient. After showing, by numerous quotations from standard authorities, that this use of cold is recognized, but not recommended, he cites from cases in his practice where its use was followed by positive beneficial results.

The method of application recommended is by bladders half filled with broken ice, to be removed as soon as they cease to be agreeable.

"The modus operandi is, no doubt, to abstract heat, to benumb exalted sensibility, and to contract the dilated and semi-paralyzed vessels; and its especial effects have seemed to be to diminish abdominal tension, to control the volvulus writhings of the bowels, and thereby to relieve both pain and tenderness."—*London Lancet.* J. S. K.

3. *Injection of Quinine in Gonorrhœa.* Radha Nauth, Roy. Assistant Surgeon. (*Indian Med. Gazette.*)

N. says: "I have invariably found the disease yield, both in its acute and chronic stage, under its influence. It acts as a tonic and astringent to the mucous membrane of the urethra. I have also used it in some cases of cystitis with much benefit. I generally use it dissolved in dilute sulphuric acid, mixed with rose water. Two grains of sulphate quinine dissolved in eight or ten minims of dilute sulphuric acid, and mixed with an ounce of rose water — to be used twice for injection. At the same time I give copaiba mixture to my patients."

"In almost all cases I have found it acts like a charm. The disease is generally cured within a week, but chronic cases take a longer time."—*Nashville Journal*, *Aug.* 26. J. S. K.

4. *A New Therapeutic Action of the Salicylate of Soda.* C. F. Kunze. (*Deutsche Zeitschr. f. Pract. Med.; Allg. Med. Central Ztg.*, 59, 1876.)

The author has tried the salicylate of soda in two cases of gout with so excellent a result that he is tempted to say: "We have in the salicylate of soda a remedy which, within two or three hours, relieves the gouty patient of all pain. The two cases upon which I base this assertion belong to the regular true gout. Every spring the patients had an attack which lasted from two to three weeks. Both patients, one sixty-seven years, the other forty-five years of age, exhibit the typical picture of a gouty person. They are plethoric, well fed, and they love wine and beer."

The older patient is the proprietor of a hotel, who has had the podagra since fifteen years. The balls of the great toes are thickened and the calcareous deposits can plainly be felt; the ankle joints also are thickened. The attack of the gout regularly returned every year; the most intense pain lasted continuously from six to eight days, then it gradually subsided, and after four or six weeks all painful sensations had left the patient so that he could go out again. The last attack began three weeks ago. The younger patient has been subject to gout since five years. Three years ago he had an attack of apoplexy with hemiplegia of the right side, from which he recovered so far that he can use his fingers for coarse work. The podagra visited him regularly every year about Easter; its last visit was three weeks ago. The author had attended him in all previous attacks of the gout, but was not called upon the last time until the most terrible pain had continued already for two days.

Each patient took one drachm, (four grammes,) of the salicylate of soda in one dose. After three hours there was not a sign of pain, but they were still red and glistening as before. The whole body was covered with a profuse perspiration. The second patient had tinnitus aurium, and a dullness of hearing to such a degree that he could scarcely understand a word. Both patients slept uninterruptedly the whole night; on the next morning no spontaneous pain, but pressure on the swollen joints and attempt at walking occasioned a slight soreness. The noises in the ear disappeared during the morning; appetite good, thirst slightly increased, pulse quiet, urine scanty and highly colored. For ten days each patient received half a drachm, (two grammes,) of the remedy and was strictly prohibited from using the affected limbs. The pain did not return, the sleep was undisturbed; only the swelling of the balls of the feet persisted, but receded readily under the mild pressure of a flannel bandage. Besides the above remedy the patients took a daily draught of Friedrichshaller bitterwater for the constipation.

5. *The Alcohol Dressing* BOSLEE. (*Bulletin de l'Acad. de Méd. de Belgiqne* T. x. 1876.)

The author prefering this dressing to that recommended by Lister, employs simply a camphorated alcohol at 20 degrees. Charpie saturated with alcohol, is simply laid over the parts where there is solution of continuity, in ordinary wounds, or those following amputation. If reunion is impossible, pledgets of the lint are insinuated between the lips. Over all is applied a compress and bandage. Before proceeding to bring the parts together, it is well to use the alcohol as a lotion over the entire surface. An open suppurating wound is filled with the charpie saturated with alcohol, care being taken to see that all sinuosities are kept dilated by the same means. A piece of rubber or oilcloth will prevent evaporation. If the wound is large, the dressing should be renewed at night, and the alcohol should be then diluted with water in order to avoid the delirium which is doubtless occasioned by the absorption of the liquid. But this latter accident never occurs upon a granulating surface. The alcohol favors immediate union. In obliterating the vessels of small calibre, whose orifices communicate with the surface of a wound, accumulation of blood is prevented in the deep tissues—an occurrence which often prevents union by the first intention. Inflammation is thus also prevented. Slight compression is often necessary to obtain the end in view. The wound and the dressing never emit a disagreeable odor; the latter is simple, clean, and readily applied; is never painful, and prevents the pus from becoming putrid.

6. *Toxic Properties of Glycerine.* DUJARDIN-BEAUMETZ. (*LaFrance Médic,* No. 62.)

The author's researches were conducted in view of the tendency which exists to employ glycerine internally in large doses, as for example in diabetes. According to Bertholet this substance is a triatomic alcohol, and should therefore produce effects identical with those produced by alcohol. The conclusions are as follows:

1. Chemically pure glycerine, when introduced beneath the skin of a dog in doses from eight to eighteen grammes per kilogramme of the weight of the animal, will determine fatal symptoms in twenty four hours.

2. The ensemble of toxic effects, (acute glycerism,) is, within certain limits, comparable to those of acute alcoholism.

3. The necroscopic lesions in acute glycerism are analagous to those of alcoholism, which leads to the conclusion that the toxic action of the two bodies is almost identical.

4. In a therapeutic point of view, the introduction into the economy of large quantities of glycerine is not without danger.

We should therefore employ glycerine internally with caution, and not lose sight of the fact that glycerine, like alcohol, can produce favor able or unfavorable results, according to the dose and mode of its administration.

7. *Air as an Anæsthetic.* DR. P. D. KEYSER. (*Phil. Med. Times*, July 22, 1876.)

K. speaks very highly of air as an anæsthetic in small minor operations. He says: It is to my mind one of the simplest, and at the same time one of the most beneficial agents in small operations about the eye that have been presented to the profession; its application being very easy, requiring no recumbent position on the part of the patient, calling for no apparatus for its administration, and being perfectly free from any of the disagreeable effects of ether or chloroform.

To produce the proper effect the patient must open his mouth, breathe freely, quickly and deeply, and after a few seconds or minutes of such steady, continuous breathing, the symptoms of partial anæsthesia supervene, as is evidenced by the absence of feeling on pinching or pricking with a pin. The anæsthetic effect passes almost immediately away, and the patient feels no pain in the operation if done dexterously and without hesitation.

Any one interested in the subject I would refer to an article by Dr. Bonwell, on "Air as an Aesthetic," in the *Pennsylvania Journal of Dental Science.—New York Med. Journal*, Sept., 1876. J. S. K.

8. *The Combined use of Morphia and Atropia in Spasmodic Asthma.* OLIVER. (*The Practitioner*, February, 1876.)

The use of the combination of morphia and atropia, by sub-cutaneous injection in spasmodic asthma, Dr. Oliver maintains are superior to morphia alone, in that their good effect is more speedy and complete, and that they produce no gastric disturbance, that they can be safely used, very frequently without injury to the general health, both as a prophylactic and as a curative means, and that the injections are followed by very speedy relief, five, and at the farthest, twenty minutes being sufficient to produce relief.—*Journal of Nervous and Mental Diseases*, July, 1876.

